# The role and contribution of experts by experience in building research capacity in adult social care services: findings from the script study

**DOI:** 10.1186/s40900-025-00779-z

**Published:** 2025-08-25

**Authors:** Krystal Warmoth, Leisha O’Brien, Ana-Maria Bilciu, Jeremy Dearling, Jennifer Lynch, John Woolham, Eneida Mioshi, Kathryn Almack

**Affiliations:** 1 Wolfson Institute of Population Health, Queen Mary University of London, Mile End Road, London, E1 4NS UK; 2 Academic Centre for Healthy Ageing, Barts Health NHS Trust, London, UK; 3 University of Hertfordshire, Hatfield, UK; 4 Shaping Our Lives, London, UK; 5 King’s College London, London, UK; 6 University of East Anglia, Norwich, UK

**Keywords:** Public involvement, Service user involvement, Social care research, Co-production, Participatory approach

## Abstract

**Background:**

Engaging individuals with lived experience in social care research is crucial for transforming how knowledge is generated and applied in practice. This study explores the involvement and perceptions of experts by experience in the SCRiPT study, which aimed to build research capacity in social care through Research in Practice Teams (RiPTs). These teams included social care professionals and experts by experience who contributed their lived expertise to research design and execution.

**Methods:**

A qualitative approach was used to analyse data from semi-structured interviews and focus groups with RiPT members, social care staff, and experts by experience.

**Results:**

Despite the challenges of recruiting experts by experience into social care research, it was found that experts by experience made significant contributions, offering unique perspectives based on their personal experience with health and social care services. Their input improved the relevance of research, helped shape study design, and ensured the research was more applicable to practice. They also brought valuable research experience, with some contributing to data analysis and ethical discussions. Furthermore, their involvement added enthusiasm and energy to the teams, inspiring greater engagement. Efforts to ensure accessibility and inclusivity were made, including clear communication, adjustments for different impairments, and additional support from a user-led organisation. Experts by experience were seen as equal partners, and their involvement led to positive changes in team dynamics and attitudes. Initially, some team members were unsure of the experts’ role, but later interviews revealed a shift in perspectives, with many recognising the contributions.

**Conclusions:**

This study underscores the positive impact of involving experts by experience in social care research. It highlights the potential for co-production to enhance research capacity, improve service delivery, and influence future research practices. Findings suggest that a participatory, collaborative approach can lead to meaningful involvement and improved outcomes, reinforcing the need for experts by experience to be recognised as essential, equal contributors in research teams.

**Supplementary Information:**

The online version contains supplementary material available at 10.1186/s40900-025-00779-z.

## Abstract (plain language summary)

People who work in adult social care want to make sure their services are based on good evidence. But in the past, there hasn’t been much money or the ability to do research. To help change this, a project called ‘SCRiPT’ (which stands for Social Care Research in Practice Teams), led by the University of Hertfordshire, worked with two local councils in the East of England.

In this study, four small research teams were set up. Each team was led by a social worker or occupational therapist. These team leaders got help and training from university researchers to do studies in their own workplaces. Each team included social care staff and people who have used social care, also called Experts by Experience, or EbEs. Some were already working for the council, and others were brought in by an organisation called Shaping Our Lives, which helps make sure everyone has a voice. This group also supported the EbEs throughout the study.

During and at the end of the study, the people in the research teams asked what they thought about involving EbEs in the teams. Most people said it was a really good experience. How much each EbE was involved depended on things like how well they knew them previously and whether they had done research before. The study showed that it’s important to include people with lived experience. It also showed they can be equal partners in research. This is a step forward for future involvement work in social care.

**Supplementary Information:**

The online version contains supplementary material available at 10.1186/s40900-025-00779-z.

## Key Message

Experts by Experience (EbEs) enriched social care research by contributing lived knowledge, enthusiasm, and in some cases research expertise, enhancing project quality and relevance. Early recruitment, appropriate support, and inclusive practices enabled meaningful participation and influence on research design. Involvement had lasting impacts, with professionals reporting changes in practice and EbEs gaining skills and confidence. Challenges included late recruitment, resource demands, and underrepresentation of marginalised groups. Findings highlight the need for early, equitable, and diverse involvement to ensure EbEs are recognised as essential and equal partners in social care research.

**Supplementary Information:**

The online version contains supplementary material available at 10.1186/s40900-025-00779-z.

## Background

Involving people with lived experience in social care research is a meaningful shift in how impactful knowledge is created. Their personal experiences, or ‘lived expertise,’ bring unique insights that add depth and real-world understanding to research. Service users and carers are now seen as credible contributors, helping shape studies at all levels [1]. Their involvement improves the quality and relevance of research by adding perspectives that professionals alone may not offer and is recognised as contributing to good practice in health and social care in the UK [2]. Inclusion of members of the public who have lived experience of drawing on social care services is valued within research studies for their contributions of their own, original, experiential knowledge, which adds new dimensions to knowledge construction, and has enhanced both the quality of the research and the impact of its outcomes [3]. Over time, this has led to more inclusive approaches, including co-produced and service user-led studies, making research more connected to the needs and realities of those who use services.

There is a growing requirement from social care professional bodies to include the voice of service users and members of the public, as active members of research teams (such as advisors, collaborators and co-investigators) [4]. Despite growing recognition of the value of lived experience in research, user and public involvement in social care research faces unique challenges such as the lack of an established way to reach people who use social care services—unlike in health, where patient forums often exist—and the fact that social care staff are less familiar with working alongside service users as equal partners in research or evaluation [5]. Barriers, such as power dynamics, lack of funding, and limited awareness of co-production methods, hinder active participation [6–8]. Despite growing recognition of the value of involving people with lived experience in research, there remains a significant lack of research on social care involvement, along with major challenges to meaningful involvement [9].

It must be noted that the language used to describe public involvement in social care research is widely debated and often contested [10] and was a topic of debate for this paper amongst the authors. Terms such as ‘service users’, ‘people who draw on social care services’, ‘experts by experience’, and ‘lived experience partners’ are all used, each carrying different meanings and emotional responses. Some individuals are viewed as ‘professionals’ rather than members of the public, either because of their prior roles or their long-term involvement in research, which raises questions about the value of their lived experience, the meaning of public involvement, and whose voices are truly being represented [11]. In the SCRiPT study, we have encountered strong opinions both supporting and opposing the use of these terms, reflecting the complexity and sensitivity of identity in this context. For this study and paper, we have adopted the term ‘experts by experience’ (EbE) as this is still common terminology that refers to people who have personal experience of using or caring for someone who has used social care services. Members of the public involved in the present study included people with lived experience of drawing on social care services, informal carers, and some who had had previous involvement in social care research, so the term was deemed appropriate, and the term was most agreeable amongst those who contributed to the study.

This paper reports on the involvement of EbEs in the SCRiPT (Enhancing research capacity in adult social care and social work in the East of England: testing the feasibility of *S*ocial *C*are *R*esearch *i*n *P*ractice *T*eams) study. The overall aim of the SCRiPT study was to test ways to build research capacity in social care using research in practice teams (RiPTs) [12]. The RiPTs comprised social care professionals (one Lead and up to four Associates) and up to three EbE team members. Each RiPT developed and undertook a small social care research project over two years. A crucial aspect of building research capacity and capability in the SCRiPT study was supporting the social care professionals in the RiPTs to learn how to effectively involve EbEs in the research process. Most EbEs (8 of 10) were introduced to RiPT Leads by Shaping Our Lives (SoL), a user-led organisation that specialises in inclusive involvement, but in one RiPT, two EbEs were already employed by the local authority taking part in the SCRiPT study and were colleagues of the RiPT Lead. EbEs and RiPT Leads were supported by SoL throughout the SCRiPT study.

## Methods

The present study utilised the qualitative data collected as part of the larger evaluation of the SCRiPT study to explore the perceptions and experiences of working with EbEs (see Table 1). This data was collected from semi-structured interviews with RiPT Leads (*n* = 4) conducted approximately 6 months after the establishment of the RiPT teams and again when the study ended. Data from RiPT Associates were collected via 4 focus groups (*n* = 9), conducted approximately a month after joining the RiPTs, and the Associates were then individually interviewed at the end of the study (*n* = 10). Additionally, semi-structured interviews with other key stakeholders were conducted when the RiPT teams were being set up (*n* = 17) and again when the study had finished (*n* = 9). These individuals were selected because they had indirect connections with the SCRiPT study, as managers or colleagues of RiPT members. They were well-positioned to offer valuable insights into the wider functioning and impact of the RiPTs. All interviews and focus groups were conducted by an experienced qualitative researcher with support from other members of the project team. Topic guides sought to explore views and experiences of research use in social care; perceived barriers and facilitators to research use, and views on the impact of EbE contributions. All interviews and focus groups by the research team were audio-recorded and transcribed for analysis. Interview and focus group participants provided written consent for participation and publication of findings. The informed consent exchange was also audio-recorded, and participants were asked to audibly agree to each part of the process.

**Table 1:** Data collection from script used in the present study

Type of data collected					
**Timepoints**	Baseline / Midpoint			Endpoint (after study ended)	
**Participant data**	Semi-structured interview	Focus group	SoL informal session	Semi-structured interview	SoL informal session
RiPT leads	*N* = 4 (6 months after RiPT)		*N* = 4 (3 months after EbE joined RiPTs)	*N* = 4	*N* = 4 (1 year after joining RiPT)
RiPT associates		*N* = 9 (1 month after RiPT)		*N* = 10	
EbEs			*N* = 9 (3 months after EbE joined RiPTs)		*N* = 10 (1 year after joining RiPT)
Other key informants/ stakeholders	*N* = 17 (during RiPT setup)			*N* = 9	

Additional data from the EbEs about their views and experiences in the RiPTs was collected by the service user involvement partner organisation (SoL), as this had not been included in the original study protocol. The primary objective of these one-on-one sessions was for SoL to engage with the RiPTs, particularly the EbEs recruited by SoL, to ensure they were supported to reflect on their participation, offer feedback, and identify any challenges that SoL could work to address and resolve. Two one-to-one informal sessions with EbEs (*n* = 9 and 10, respectively) and RiPT Leads (*n* = 4) were conducted by SoL approximately 3 months after EbEs had joined the teams and after a year’s involvement. They were asked about their experience as EbEs or the involvement of EbEs and contribution to the teams, facilitators and barriers to involvement, learnings from the experience, and any support that was needed. These sessions were not audio-recorded or transcribed; however, notes were taken by SoL staff to record reflections about people’s experiences, together with reflections from SoL about the role of these short one-to-one sessions. Informed consent was obtained retrospectively to allow the notes from these sessions to be included in the analysis.

For the present study, a targeted search of the overall evaluation data for references to EbEs was undertaken. These findings are therefore a targeted subset of the wider evaluation and are intended to illustrate how EbEs were involved and the impact of their contributions. The first and second authors extracted and analysed data relevant to EbEs; then the data were collated and summarised for each RiPT. A practical thematic analysis approach was adopted to analyse the data [13]. Researchers [KW and LOB] listened to each recording, read and reviewed the data to familiarise themselves with it, then wrote memos, defined as brief notes on thoughts and questions arising during reading, and a summary of their impressions of the dataset. Descriptive full thought codes were assigned and used to construct meaningful broader themes by the first author, and the themes were discussed with the team to enhance reflexivity. Comparisons across the different RiPTs were noted throughout the analysis. Any discrepancies were solved by discussions and coming to a consensus with experienced peers within the research team. Researchers used Microsoft Word to manage the data.

We acknowledge that our positionalities—shaped by our professional roles, prior experiences, and personal connections to the research topic—influenced every stage of this study. Comprising qualitative researchers, experts in social care research, experienced involvement facilitators, and an EbE representative, the team brought diverse professional and lived experiences; each holding different forms of knowledge and power within the research process. Reflexive practices, including team reflections, feedback loops, and collaborative analysis, were used to challenge assumptions. Despite these efforts, we acknowledge that the findings are co-constructed and reflect the interplay between participants’ accounts and our interpretive lenses.

## Results

Overall, there were positive views about the involvement of EbEs in the RiPTs. Key themes related to how EbEs joined the RiPTs, how they were introduced and welcomed into the RiPTs, factors that supported their involvement and participation in the RiPTs, their various contributions, and the lasting impact of this experience.

### The role of recruitment processes in shaping EbE participation

Recruitment––and thus the initial level of participation─differed to some extent between the EbEs who were already employed by local authority and those who were recruited into the RiPTs by SoL. For example, in RiPT 3 (see Table 2), their local authority employed EbEs, who provided input on service provision and delivered training on accessibility, so they were known to the RiPT Lead and other team members. Consequently, they contributed from the start of the RiPT and therefore were integral to the formation of the research question and the design and delivery of the study. The RiPT members, who work with people with lived experience employed by a local authority to contribute their experiential knowledge, did not question or express concern about how the EbEs would contribute, or if they could participate. The Lead had previous experience of involving EbEs and was used to adjusting how they worked and taking steps to ensure that the EbEs were included.

**Table 2:** Details of RiPT and expert by experience involvement

	RiPT 1	RiPT 2	RiPT 3	RiPT 4
**Study topic**	Exploring the lived experiences of older people discharged via ‘Discharge to Assess’ Pathway One, i.e. people who are discharged to their own homes	Exploring the effectiveness of the Morriston Occupational Therapy Outcome Measure (MOTOM) tool in reablement services.	The impact of occupational therapy in adult social care learning disability services	Exploring the use of Technology Enabled Care (TEC) digital solutions to support the welfare and independent living of their most vulnerable citizens
**Total number of Experts by Experience**	3	2	3	3
**Employed by local authority**	0	0	2	0
**Experience of study topic and research involvement**	Direct experience with topic (2); indirect experience as carer (3)	Experience of NHS services (1); direct experience with topic (1); research experience (1)	Experience of social care services (3)	Experience of social care services (2); research experience (2)

NB. Figures in brackets denote the number of EbEs and their experience level with the study topic and research

*I think because not just about time*,* but about the accessibility of information and the time required to develop that in the first instance*,* I think…But*,* for me*,* I’ve had separate times where I’ve had chats with [EbE]*,* for example*,* to go through something that he may not have been able to understand as easily through an email*,* or something like that…* [RiPT 3 Lead, first interview].

The employed EbEs also had more availability for meetings and discussions outside of the scheduled RiPT meetings to consult and prepare for the upcoming meetings, therefore influencing the extent to which they were engaged.

Social care professionals in the other RiPTs were not experienced in working with EbE, as the Lead was in RiPT 3. Initially, they expressed doubts about the value of this involvement and had concerns about the potential contributions of the EbEs, especially as the externally recruited EbE joined later. One Associate discussed how they had initially questioned whether EbEs were needed, as the team had already determined the topic and research question [12].

*Well*,* I think as it gone past the point of like we’d… come up with what we were doing anyway and then it was sort of put to us all. Do you think we need them involved or not?* [RiPT 2 Associate, second interview]

However, these views in the RiPTs had changed, and the contributions of EbEs were viewed more positively.

*I was a bit apprehensive to begin with*,* as to how it would impact the team*,* if the [practitioners] would still be truthful and honest. That has not been a problem at all – [EbEs] have been really helpful*,* in different ways.* [RiPT 2 Lead, second interview]

For those RiPTs that had to recruit EbEs, Leads and Associates observed that they would have benefitted from the involvement of EbEs from the start, discussing how they thought that research was better when it is co-produced throughout the entire project.

Most of the RiPTs recruited EbEs through an external service user involvement partner organisation (SoL) to identify and recruit EbEs who had experience with their individual research topics, such as the Discharge to Assess pathway (discharged to home) or reablement services [12]. Leads were directly involved in the recruitment process of EbEs for their RiPTs so that it was done in a collaborative manner, to ensure relevant experiences. Recruitment proved challenging, as there were no established social care service user forums comparable to those in healthcare (e.g., patient forums), making it difficult to locate individuals with direct experience of the relevant services. Furthermore, since EbEs were recruited through an external organisation and some topics were highly specific, their knowledge and expertise did not always directly align with the focus of each RiPT (see Table 2).

### Engaging and supporting EbEs

A key theme was about the ways that the EbEs were supported and engaged so they could have an effective role in the RiPTs. This included not only considering different impairments (e.g. sensory impairment and disability) and making practical arrangements (e.g. online meetings so they would not have to travel), but also changing the way social care professionals talked in the meetings (e.g. avoiding jargon) and ensuring clear communication about what EbEs were being asked to do (e.g. obtaining feedback on study materials). For example, when working with EbEs who had sensory impairments, the CoP had to ensure that their practical arrangements for meeting and working as a CoP were inclusive and accessible—this meant, in virtual meetings, onscreen material needed to be read aloud, and written materials were provided in accessible formats. Leads described how they worked to overcome barriers to participation to facilitate inclusive and accessible practices.

In addition, SoL undertook a ‘bridging’ role to support involvement, which included conducting one-to-one informal sessions with both EbEs and Leads. One person stated how they appreciated this additional support for their RiPT meetings.

*I think it was a great addition and I can see how perhaps when we were thinking about supporting people and ensuring that they’re equipped to actively contribute in these meetings and the one to ones really played into it….people reflecting on their experiences of being involved and kind of finding their way through being able to vocalise what they’ve learned*,* how they’ve grown. And what kind of impact they’ve had*… [EbE, informal session].

As most Leads were inexperienced in research involvement, SoL facilitated an introductory session for all Leads in which SoL provided an overview of the practicalities of working well with service user representatives, which took place ahead of EbEs officially joining their teams, and after most recruitment activities concluded. This additional support seems to have facilitated collaboration, and EbEs recognised and appreciated these actions and efforts to include them in the RiPTs. Actions like sending out agendas, information, questions and lists of future meetings beforehand, as well as regular communication, enabled them to meaningfully participate. EbE reported how they were given the space and opportunity to contribute in various ways, were able to challenge and disagree with the team, and were fully informed so they could contribute. As one EbE reflected on the experience with the Lead:

*The good thing about [Lead] is that they talk to you as your equal – you have timely conversations during meetings as they take in your input in real-time. In terms of contributions from a lived experience*,* they do take it on – there are no barriers to contributing*,* which is superb. [RiPT 4 EbE*,* informal session]*

EbEs discussed how they felt engaged, respected, valued, welcomed, supported and confident about being a member of the RiPT.

There was a strong desire within each RiPT that there should be a ‘level playing field’ so EbEs could play a full and effective role, and for their views to be listened to and considered.

*[E]veryone [in the RiPT] has an equal voice and is hoping to achieve the same aims.* [RiPT1 Associate, second interview]

Working in RiPTs enabled practitioner members to reflect on the nature of their interactions in their normal practice, in which there was a clear imbalance in power [14]. There was a recognition that this relationship in the RiPT was different, and they attempted to address this imbalance.

### Contributions of EbEs

EbEs contributed to the RiPT in a number of ways; although employed EbE joined earlier than the externally recruited EbE, both made significant and impact contributions to their RiPTs. They brought their knowledge and lived experience as service users. This was often a new or different perspective of the study that team members who were social care professionals may not have otherwise considered or contributed in ways that made the study more applicable to practice. One Lead reflected:

*It would be a very different project without them – it would be one of my own making*,* but less relevant to what people actually want*. [RiPT 3 Lead, informal session]

EbEs made the teams consider accessibility and how they communicated with service users in team meetings.

One unexpected and valuable contribution within the SCRiPT study was the research experience brought by some EbEs. Unlike the common perception that research expertise is primarily held by academic or professional practitioners, several EbEs had previously engaged in research activities, including participation on advisory committees, involvement in research teams, and familiarity with a range of research processes and methodologies. This was in addition to bringing their own lived experience to the RiPTs. For instance, one EbE played an active role in supporting the RiPT Lead with managing and analysing qualitative data, demonstrating skills often assumed to be exclusive to formally trained researchers. As one RiPT Lead stated,

*I’ve got one of my experts by experience. He’s got some first-hand experience of doing that sort of stuff…* [RiPT 4 Lead, second interview].

This taps into debates about whether members of the public who get involved in research studies long-term lose the ability to bring an ‘outsider’ type of perspective. However, in this RiPT team. this dynamic challenged traditional assumptions about ‘expertise’ and disrupted hierarchical notions of knowledge within the team. It also highlights the value of combining EbE who have previously gained knowledge and skills about doing research alongside those newer to the role.

The overall aim of the SCRiPT study was to build research capacity within social care, a sector where many RiPT Leads and Associates initially had limited or no prior experience in research planning or execution. In this context, the EbEs’ existing research experience proved particularly useful, and it places value on their capacity building. This unique positioning enabled them to contribute in diverse and impactful ways, including reviewing study materials to ensure clarity and relevance, providing informed advice on ethical considerations, assisting with the analysis and interpretation of qualitative data, and playing a leading role in disseminating findings (such as contributing to academic publications). As one Associate described:

*He had quite like an eye for things. And he obviously knew a lot of*,* like being involved in research before*,* whereas obviously the rest of us didn’t so much. So yeah*,* he was able to bring a different perspective*,* so it wasn’t even necessarily a perspective on being like a client or on the other end of the service*,* which I guess probably was the original idea of them being involved. I mean that side was helpful*,* but it was*,* yeah*,* a lot of it was to do with his sort of more like academic research background and having involvement in things before.* [RiPT Associate, second interview]

This example highlights the diversity and fluidity of roles within collaborative research teams. Rather than adhering to rigid role definitions (i.e. EbEs can only contribute their lived experience of services), the SCRiPT study illustrated how EbEs can bring unique expertise that complements, enhances, and sometimes exceeds that of traditional practitioners or researchers. This interplay enriched the research process, fostering more inclusive, reflexive, and rigorous approaches. Importantly, this account underscores the varied and dynamic ways in which EbEs can meaningfully contribute. Their involvement demonstrates that expertise is multifaceted and that research teams benefit from embracing diverse forms of knowledge and experience, ultimately improving the quality and relevance of research outcomes.

Leads and Associates reported that the EbEs added an ‘extra energy’ or were inspiring to other team members.

*I think it has helped for me in making it more exciting – we are talking to people that want to help and it has helped me been more energised…and I felt more of a buzz about the research*,* as they have been involved.* [RiPT 1 Lead, informal session]

EbEs were perceived to be keen to contribute and very engaged. Leads mentioned how they were often quicker to respond, contributing not only their lived experience and knowledge but also their enthusiasm.

EbEs discussed how it felt good to impart their lived experience of the research topics and contribute to the teams. One EbE reflected that they initially were unsure about their contribution, but this was something that the RiPT would work collectively to help them contribute successfully:

*if my contributions aren’t making a difference then that is for them to own and communicate it to me. The relationship is*,* I believe*,* transparent enough for that to happen [RiPT2 EbE*,* informal session]*.

But, when asked later, the same EbE reported how their experiences had helped steer the team and even mitigate potential future issues with the research. Another EbE described how they thought the RiPT Lead had learned a lot because of their involvement.

*I think of [Lead] as a little sponge as they have absorbed a lot of the information we have shared with them and they have absorbed it really well … I think that [process] is incredibly powerful. [RIPT 3 EbE*,* informal session].*

Largely, EbEs viewed their involvement positively and their contributions had made a considerable difference to the RiPTs and each study.

### Lasting impact

EbEs also had a lasting impact beyond the RiPTs. As a result of the experience with EbEs in SCRiPT, many participants reflected on how this collaboration had reshaped their understanding of meaningful involvement and expressed a strong intention to include EbEs in future research and service development initiatives. Leads and Associates described gaining a deeper, more practical understanding of service user and public involvement in research, moving beyond a procedural or tokenistic approach (a false appearance of inclusiveness). They reported feeling more confident and motivated to embed lived-experience voices earlier and more consistently in the research process. One Associate explained how their experience with EbEs in SCRiPT influenced their practice in their current clinical role, shifting the way they engage with service users:

*I was more aware of how do you feel about such equipment like this and just get people talking even though it wasn’t like a formal interview. It will just be a discussion as part of my assessment…So I was actually applying that from the research in my current role [in SCRiPT]… I probably won’t even thought to ask.* [RiPT 4 Associate, second interview]

This example illustrates how the EbE collaboration not only influenced research practices but also extended into day-to-day clinical interactions, reinforcing the lasting and practical value of lived-experience input. Another Associate discussed how their experience working with the EbEs would inform their practice by changing how they communicated with service users in their frontline role.

*And also how we wrote things*,* how we presented things was …also very useful*,* not only useful for the project*,* but you know wider*,* it’s always good to talk to people who use our services.[RiPT 1 Associate*,* second interview]*.

All RiPT members also recognise why user involvement matters.

EbEs echoed these positive views, reporting that their involvement in the study helped them gain greater knowledge of the research topic and a deeper understanding of the challenges and need for research in social care. For example, one EbE reflected on learning about the complexity of research and the significant amount of work it involves. Another highlighted the importance of teamwork, saying:

*I have learned to appreciate the team around you*,* the real high level of trust that it takes – [Lead] asked me to do the work because they trusted and appreciated my work.* [RiPT 3 EbE, informal session]

In some cases, EbEs’ contributions extended beyond the life of the project. For instance, one EbE remained actively involved in dissemination activities after the two-year project concluded, making a valuable contribution to the development of an animation illustrating the RiPT’s study findings. This individual also established connections with other research initiatives and continues to play an active role as an EbE in social care research. Overall, EbEs described the experience as an opportunity for mutual learning between social care professionals and themselves.

## Discussion

The present study explored the perceptions and experiences of the EbEs in the research in practice teams (RiPT), as part of the larger SCRiPT study. The involvement of EbEs in the RiPTs was widely seen as positive by RiPT Leads and Associates, as well as by the EbEs themselves. Efforts were made to ensure accessibility and inclusivity, fostering collaboration. EbEs contributed their lived experience, research skills, and enthusiasm, enriching the teams and their studies. Initially, some Leads and Associates questioned the role of EbEs, but perspectives shifted, with all recognising the value of EbE contribution. These findings corroborate the ideas of how EbE are valuable, credible contributors to research [1–3]. The impact of the Experts by Experience (EbEs) extended beyond the RiPT project, potentially influencing future research approaches and frontline practice, while also deepening the EbEs’ understanding of social care research. Our findings are consistent with previous evidence on the impacts of patient and public involvement and engagement, highlighting not only how involvement can shape research itself, but also its wider benefits—such as shifts in perspectives, improvements in service, and the growth of involvement activity more broadly [15].

Participation differed between EbEs employed by the local authority and those recruited externally by Shaping Our Lives, with the former being involved earlier, having existing relationships with the team, and contributing more fully to shaping the research. In contrast, externally recruited EbEs joined later. For those recruited externally, relationships had to be built with the RiPT Leads and the other social care practitioners in the RiPTs. Delays in recruiting the EbE meant that the RiPTs missed out on early input into shaping the research questions. These dynamics highlight the central role of relationship-building in effective collaboration. As existing literature emphasises, successful and meaningful PPIE relies on the development of trust, mutual understanding, and clear expectations between contributors and researchers [8, 16, 17]. Furthermore, collaborative research requires attention to power-sharing, ensuring that EbEs are not just included but have real influence over decision-making [16], avoiding tokenistic or devaluing EbE input [18, 19]. In our study, the later involvement of externally recruited EbEs limited their early influence on research design, illustrating how delays and insufficient groundwork can hinder power-sharing and full participation. Nevertheless, with the support of SoL, many of these challenges were overcome. As relationships developed, the EbEs and social care practitioners established productive working partnerships, and the contributions of EbEs were recognised and valued. This aligns with findings from previous studies and recommendations showing that with adequate support and time, strong and equitable collaborations can emerge, even when initial conditions are less than ideal [4, 8]. A key challenge in public and user involvement activities is finding individuals with the capacity to participate who also have direct experience of the research topics. For example, the research topic for RiPT 4 was understanding older people’s perspectives on sharing their Activity Monitoring Data with Social Care Services. Older people in receipt of social care services are likely to be people with complex and multiple impairments who are also on lower incomes - able to meet the means-tested eligibility thresholds [20]. They are part of the wider population of people accessing social care services who are likely to be under-represented or not heard in adult social care research, policy and service planning. The combination of factors, such as age, capacity, socio-economic status, may render people more vulnerable, and it can be challenging to access their voices not only as research participants but as EbE. Shaping Our Lives were a key co-applicant in the SCRiPT study, and we were able to draw on their expertise in reaching out to and working with people from marginalized communities, especially those facing the biggest barriers in becoming involved as EbE in research. It is also worth noting that EbE self-perceptions of marginalisation or vulnerability may not always be the same as the perceptions of others [21]. Reaching individuals who have not previously been involved in research is resource- and time-intensive. Even with SoL involvement and support, it is important not to underestimate the time and resources required to recruit EbE with relevant experience to support research projects. There is a tendency to continue engaging with already-engaged groups [22], which can limit the diversity of voices in research. Certain groups, such as Black and minority ethnic service users [23], or those with communication or sensory challenges, face specific barriers and are less likely to be heard. Evidence shows that these groups are often underrepresented in mainstream involvement initiatives, including research, despite their likely overrepresentation in terms of lived experience with social care services [24]. This disparity in participation in the present study and the wider context of social care research underscores the need to improve recruitment, support wider engagement, and alignment of experience to ensure all can contribute meaningfully to social care research [14].

When positioned as co-researchers, those with lived experience can be undervalued in research by academic researchers, given their lack of involvement in research methods training and, in some cases, this is augmented due to stigma or personal doubts about their skills, experience, or knowledge [25]. In many studies, EbEs may have limited research knowledge compared to other research team members. However, in the SCRiPT study, some EbEs had more experience being involved in research than the RiPT social care professionals – this contribution was valued and utilised within the team. Moreover, EbEs were perceived to be very engaged. Other team members who were social care professionals had less capacity to contribute due to frontline duties and limited time allocated to SCRiPT (a day per month). Leads mentioned how EbE were more responsive, contributing not only their experience and knowledge, but also their enthusiasm. This illustrates the potential of EbEs to support research capacity and construct knowledge in social care. Future work could explore how experienced EbEs can further foster research capacity in social care, such as contributing to research cultures within organisations and the development of active researchers with expertise in social care and creating an environment conducive to successful research implementation.

The SCRiPT study demonstrates how concerns and perceptions about involvement can change among RiPT social care professionals (or may be absent altogether if they have prior experience working with EbEs within their organisation). Leads and Associates discussed how the knowledge and advice that EbEs contributed were valued, and it also influenced future work and their practice —shaping their thinking, informing their methods, and encouraging more collaborative and person-centred practices in both research and frontline settings. Similarly, EbEs described the experience as an opportunity for mutual learning, benefiting both the social care professionals and them. The study offers insights into a more equitable partnership role for EbEs in research teams, as well as different ways in which equal access was enabled [4, 8]. The EbEs undertook a colleague role as supporters, collaborators, and advisors in the RiPTs. There is a need for researchers to see EbE researchers as equal partners and to consider them as a reliable component of the team, rather than simply an additional variable or complication [26], particularly those from vulnerable groups. When service users and professionals collaborate as equal partners, the barriers to active participation can be addressed [6, 7], potentially leading to service improvements.

There are some limitations to this work. The study was based on a small sample, which may limit the generalisability and transferability of the findings. While the in-depth qualitative approach provided rich insights, the perspectives captured may not fully represent the diversity of experiences and only capture the experiences of those involved in the SCRiPT study and not those from other social care research initiatives. Additionally, some participants’ responses (i.e. the informal sessions by SoL) were not recorded verbatim, which may have led to the loss of nuanced detail. Moreover, as this data was collected by an external organisation (not the project team) and was not originally included as part of the planned evaluation, we did not have control over how the data was collected or recorded; however, responses added valuable insight into the views and experiences of the EbEs in the RiPTs, so it was included and consent to use this data was obtained.

These limitations could be addressed in future research by incorporating participants from multiple social care research initiatives, not just those involved in SCRiPT, which would help capture a wider spectrum of perspectives and experiences. All data collection, including informal sessions, should be recorded verbatim to preserve nuanced detail and avoid loss of meaning, and where possible, the project team should lead or closely oversee data collection to maintain consistency, ensure methodological rigour, and align with the planned evaluation framework. Additionally, building EbE data collection processes into the research design from the outset—rather than relying on external or opportunistic sources—would allow for greater control and coherence, while still enabling the integration of valuable insights.

## Conclusion

This study contributes to the evidence base by highlighting the contributions and impact of individuals with lived experience who actively engage in research, particularly alongside social care professionals with little or no research experience. Participants in the SCRiPT study reported that the views and experiences of EbEs were successfully incorporated into team projects, and this involvement had a positive and lasting impact on the social care professionals and their studies. Findings suggest that a participatory, collaborative approach can lead to meaningful involvement and improved outcomes, reinforcing the need for experts by experience to be recognised as essential, equal contributors in research teams.

## Supplementary Information

Below is the link to the electronic supplementary material.

Supplementary Material 1

## Data Availability

No datasets were generated or analysed during the current study.
