# Supplementary material for: The role and contribution of experts by experience in building research capacity in adult social care services: findings from the script study
Source: Res Involv Engagem. 2025 Aug 25;11:103. doi: 10.1186/s40900-025-00779-z (PMC12379536; doi:10.1186/s40900-025-00779-z)
Supplement: Supplementary file 1 — Supplementary Material 1 [file 40900_2025_779_MOESM1_ESM.docx]

| **Section and topic** | **Item** | **Reported on page No** |
| --- | --- | --- |
| 1: Aim | Report the aim of PPI in the study | 5 |
| 2: Methods | Provide a clear description of the methods used for PPI in the study | 6-7 |
| 3: Study results | Outcomes—Report the results of PPI in the study, including both positive and negative outcomes | 7-13 |
| 4: Discussion and conclusions | Outcomes—Comment on the extent to which PPI influenced the study overall. Describe positive and negative effects | 13-15 |
| 5: Reflections/critical perspective | Comment critically on the study, reflecting on the things that went well and those that did not, so others can learn from this experience | 7-15 |

Supplementary File

Table S1. GRIPP2 checklist
